# Optimising a landscape visual quality protocol. A Method for reducing respondent fatigue and obtaining site-specific indicators

**DOI:** 10.1016/j.mex.2023.102455

**Published:** 2023-10-20

**Authors:** Ana Medeiros, Cláudia Fernandes, João F. Gonçalves, Paulo Farinha-Marques

**Affiliations:** a CIBIO, Centro de Investigação em Biodiversidade e Recursos Genéticos, InBIO Laboratório Associado, Campus de Vairão, Universidade do Porto, 4485-661 Vairão, Portugal; b Departamento de Geociências, Ambiente e Ordenamento do Território, Faculdade de Ciências, Universidade do Porto, 4169– 007 Porto, Portugal; c BIOPOLIS Program in Genomics, Biodiversity and Land Planning, CIBIO, Campus de Vairão, 4485-661 Vairão, Portugal; d proMetheus—Research Unit in Materials, Energy and Environment for Sustainability, Instituto Politécnico de Viana do Castelo (IPVC), Avenida do Atlântico, no. 644, 4900-348 Viana do Castelo, Portugal

**Keywords:** Scenic quality, Expert assessment, Visual assessment, In situ evaluation, Assessment protocol, Landscape studies, landscape visual quality protocol Optimization – Performance and Redundancy Test (LVQPO – PRTest)

## Abstract

Evaluation of landscape visual quality is crucial for policymaking and planning but is still challenging. A wide range of visual assessment protocols is available, but there is still no consensus on appropriate indicators or approaches. Also, evaluation protocols can encompass many indicators, being exhaustive and complex and making the evaluation lengthy. Furthermore, protocols tend to be catered to a particular type of landscape or site-specific, and it can be tricky to ensure the protocol developed is adequate for the landscape under study. This paper proposes a methodology to optimise the selection of indicators for landscape visual assessments. There are two main goals: i) reduce the evaluation time to avoid respondent fatigue, and ii) make the protocol site-specific, choosing indicators that perform better and avoiding redundant indicators.

• The presented method optimises the selection of indicators in expert visual assessments;
• Indicators are rated in situ on a 5-point scale and go through a performance and redundancy test;
• It helps to adapt complex evaluation protocols to the study landscape and to choose robust indicators in a supported and scientific way.

## Graphical abstract

**Figure:**
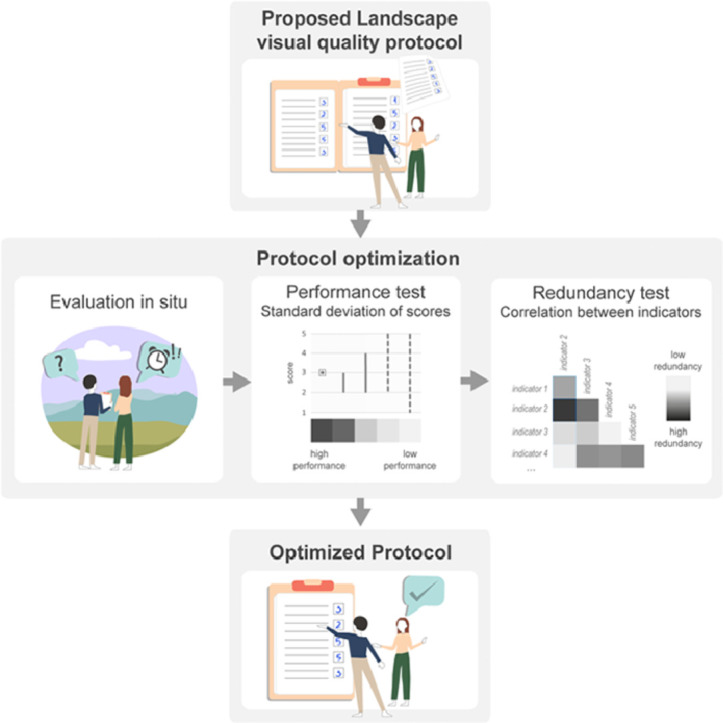

Specifications table

**Table:** 

Subject area:	Environmental Science
More specific subject area:	Landscape architecture. Landscape visual quality assessment
Name of your method:	landscape visual quality protocol Optimization – Performance and Redundancy Test (LVQPO – PRTest).
Name and reference of original method:	Visual Resource Management framework by the Bureau of Land Management [14] and [16] nine-part categorisation of 'key concepts for analysing landscape character'.
Resource availability:	Not applicable.

**Method details**

## Background

In the context of rapid and irreversible landscape changes, understanding how to evaluate landscape quality is crucial for policymaking and planning. Several visual assessment protocols have been developed, and significant advancements have been made in assessing the visual quality of landscapes [6]. However, a division between expert descriptive methods (deriving from an objective paradigm) and public preference models (deriving from an subjective paradigm) is still noticeable in landscape assessment research, inherited from the philosophy of aesthetics [4,17].

Expert assessments using an objective paradigm played an important role in the early days of landscape assessment during the 60′s and 70′s [18]. In these type of assessments, the expert evaluators would focus on biological or ecological and formal aesthetic elements, emphasising the landscape's physical structure. In contrast, the subjective paradigm has dominated the research in the last 20 years due to a growing need to involve the public in landscape matters and, consequently, an agenda shift to public opinion studies [19]. This approach is based on psychological criteria, comprising cognitive and phenomenological aspects, and focuses on the observer's interpretation of the landscape. Between the indicators used we find, for example, mystery, complexity, legibility, coherence [2,10] or naturalness, stewardship, historicity, imageability and ephemera (Ode [12]). These types of studies also analyse how the subjective views vary depending on one's age, profession, background, environmental expertise, and other social dimensions [5,7,13].

A compromise between expert methods and preference models was presented by [15], namely, the psychophysical or surrogate component models. This approach is considered more holistic for using statistical techniques to determine the mathematical relationships between physical elements in the landscape and the scenic preferences of observers [3,4,9]. For this reason, it has gained favour in research in recent years [6], as they are able to encompass a broader range of the spectrum between objectivism and subjectivism studies, adding a needed complexity between landscape elements and how we perceive them. However, there is no consensus on the procedure for evaluating landscape visual quality and the appropriate choice of indicators [9].

The proposed method improves an expert assessment where visual quality is evaluated through a landscape inventory or a visual resource assessment protocol. Using this method, experts fill in an assessment form, where several indicators are analysed, assigned a classification, and a final score is calculated. However, an optimization is frequently needed, since protocols are usually developed for a particular location, and there is no methodology to make a universal selection of indicators. Moreover, these evaluation protocols can be time consuming and complex, making the evaluation a lengthy process.

In this study, we propose a methodology that has two main goals: i) reduce the evaluation time to avoid respondent fatigue, and ii) make the protocol site-specific, choosing indicators that perform better and avoiding redundant indicators. Authors start by developing their own assessment protocol using a set of indicators suggested by two sources: one expert standard methodology by the Bureau of Land Management [14], the Visual Resource Management (VRM) program and a theoretical paper on landscape aesthetics by Ode [12].

The first one was selected for being an established methodology that has supported many frameworks developed through the years and has proven adaptable to different landscape contexts. In contrast, Ode [12] builds on the conceptual framework established by [16] and gather the nine relevant concepts from landscape aesthetic literature, including landscape preferences and perception studies, with empirically tested evidence. All these indicators are supported by concepts and theories, such as the Prospect-refuge theory by [1], Information Processing Theory by [10] or Biophilia by [11]. Recent mixed or holistic studies increasingly incorporate concepts such as those proposed by Ode, Tveit, and Fry (2008), finding them very useful in describing landscape character and visual quality.

## Methodology

### Step 1: Development of a proposal for LVQ protocol

The first step is the development of a proposed LVQ protocol. A rapid systematic review was performed in Scopus and Web of Science to identify the main expert assessment protocols used in the scientific research using the search term landscape AND (visual OR "scenic beauty" OR "visual quality" OR "aesthetic quality") AND (assess* OR eval*). The Literature search was carried out in three steps: i) identification - search in the databases and exclusion of duplicate results; ii) Screening - selection of records through the title and abstract; and iii) eligibility - selection of records through the full paper. Records were stored in EndNote 20 software, and only 545 records were expert approaches and satisfied the criteria for the Literature review. Records that did not satisfy the criteria were, for example, public perception studies, visual impact assessments of built structures, image analysis and spatial simulations, watershed pollution assessments or soil-related studies.

This literature search has enabled the selection of the two sources used to develop a proposed visual assessment methodology, and the indicators suggested in these studies were then compiled in attributes. This protocol considers three types of attributes: physical, aesthetic, and psychophysical.

**Fig. 1:**
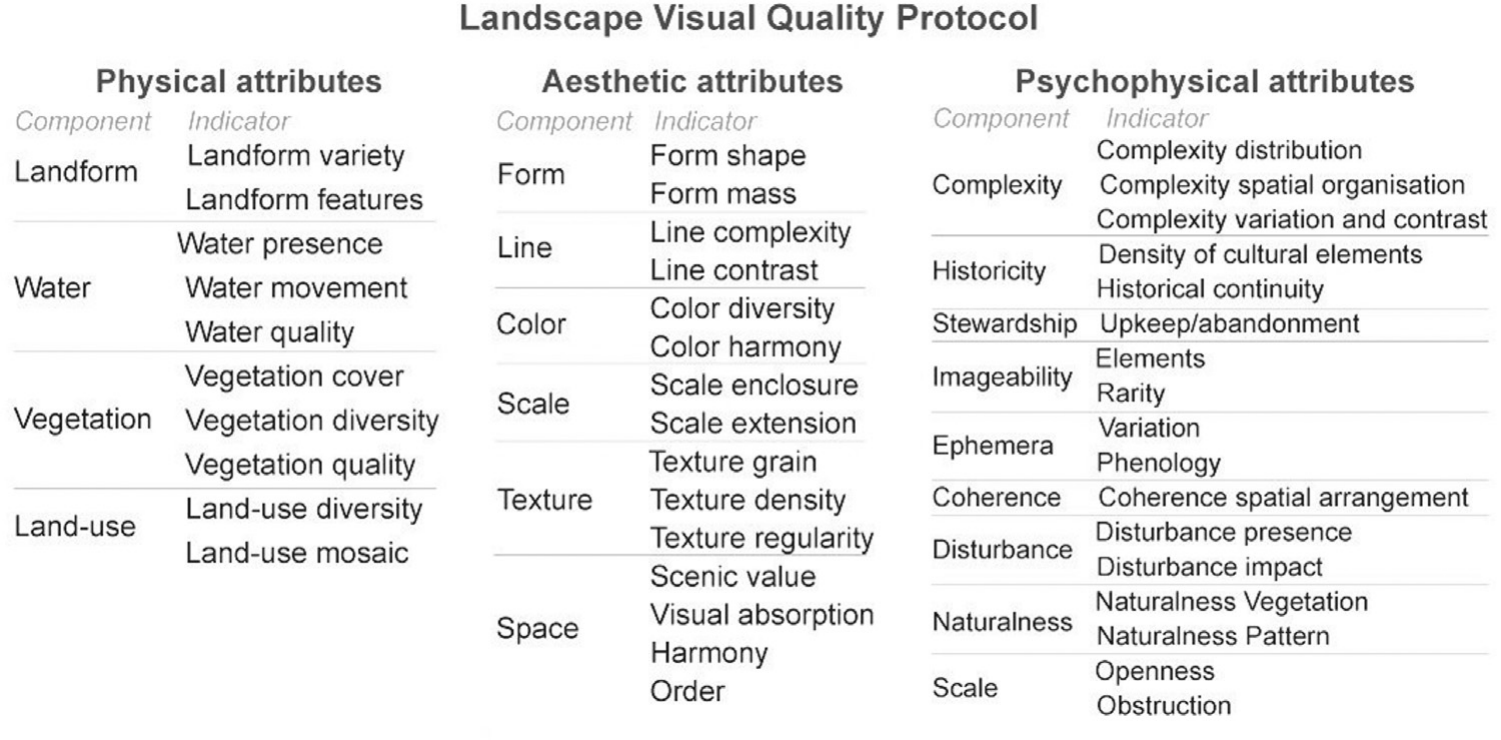
Proposed landscape visual quality protocol is divided into physical, aesthetic and psychophysical attributes, with the respective indicators for each component.

Physical and aesthetic attributes are supported by the Visual Resource Management (VRM) framework by the Bureau of Land Management [14]. The VRM program is complex as it establishes a national-level framework for the inventory, planning, and management of US public lands to maintain scenic beauty. The evaluation process in VRM consists of three steps: i) assessment of the visual quality of the landscape, ii) the sensitivity of the people to change(s) in the landscape, and iii) the viewing distance. Here, we focused on the indicators mentioned in the first step, translating them into four physical components and ten indicators, as shown in Fig. 1.

The aesthetic indicators were chosen from the same source, resulting in six components and 15 indicators. While the psychophysical indicators were based on [16] nine-part categorisation of 'key concepts for analysing landscape character', which resulted in nine components and 17 indicators.

Although the visual concepts and their visual indicators are presented independently, they are interrelated and cover a wide range of aspects of the visual landscape. For example, stewardship is usually related to naturalness, as landscapes with higher human involvement have a lower degree of naturalness. The concepts of coherence and disturbance also occupy extremes of the spectrum since the presence of elements disturbing the landscape tends to lower its consistency or coherence.

The proposed framework has 42 indicators: ten physical, 15 aesthetic and 17 psychophysical indicators. A five-point Likert scale (1 to 5) was used to score landscape aesthetic quality for each evaluation criteria. The evaluation criteria were suggested from the literature and adapted to the study area specificities when needed (Tables 1, 2 and 3). For example, the percentages of vegetation cover, the number of colors considered for colour diversity and historical periods concerning historical continuity were adjusted to the study area context.

**Table 1:** Evaluation criteria for the physical attributes.

Component	Indicator	Evaluation criteria				
		1	2	3	4	5
Landform	Variety	Low hills or flat valley bottoms	–	Some variety of vertical relief but not much surface variation	–	High vertical relief or large surface variation
	Features	Few details or no dominant or exceptional feature in the landscape.	–	Interesting slopes, valleys or erosive patterns; or characteristics that are interesting but not dominant or exceptional.	–	Prominent cliffs, rocky outcrops, eroded formations; or unusually striking or intriguing details of dominant features.
Water	Presence	Absent or present, but not noticeable.	–	Present, but not dominant in the landscape.	–	Dominant factor in the landscape
	Movement	Still	–	Flowing	–	Cascading
	Quality	Appearance not clear or clean	–	Clear and clean appearance, but without the presence of riverine vegetation	–	Clear and clean appearance and presence of riparian vegetation
Vegetation	Cover	Mediterranean forest and shrubland < 10 %.	–	Mediterranean forest and shrubland represent 20–30 % of the landscape.	–	Mediterranean forest and shrubland ≥ 50 % of the landscape.
	Diversity	Patches with little or no variety or contrast in vegetation.	–	Some variety of vegetation, but only one or two main types. No variety of shapes, textures or patterns.	–	A variety of vegetative types expressed in diverse shapes, textures and patterns.
	Quality	Dominant presence of exotic invasive species.	–	Presence of some exotic invasive species, but not dominant.	–	No exotic invasive species
Land-use	Diversity	Visual diversity and variety of land cover/use in the landscape. (More than 4 main types)	–	Some variety of ground cover. (3 to 4 main types)	–	Little or no variety or contrast in land cover/use. (1 or 2 main types)
	Mosaic	No intricate pattern, imperceptible mosaic, or low 3D structure	–	Partial presence of an intricate or mid-scale mosaic	–	Dominant presence of a small and intricate landscape mosaic

**Table 2:** Evaluation criteria for the aesthetic attributes.

Component	Indicator	Evaluation criteria				
		1	2	3	4	5
Form	Shape	Simple, indistinct, or little diverse forms	–	Shapes with medium diversity, contrast, or complexity	–	Shapes with high complexity, contrast, prominence, or diversity
	Mass	Simple, indistinct, or little diverse volumes	–	Volumes with medium complexity, contrast, prominence, or diversity	–	Volumes with high complexity, contrast, prominence, or diversity
Line	Complexity	No lines or poorly defined lines (e.g., transition edge)	–	Some evident lines of low or medium complexity (e.g., band edge)	–	Pronounced lines evident with some complexity (e.g., digitate edge)
	Contrast	Lines with little contrast or diffuse. (e.g., fuzzy edge)	–	Contrasting lines in one dimension (e.g., adjacent edge)	–	High-contrast lines between different planes (e.g., line-silhouette)
Colour	Diversity	Monochrome landscape	–	Medium diversity, with 2 or 3 main colours, low range of brightness or saturation	–	High colour diversity, various degrees of brightness and saturation
	Harmony	Dominant colours that are very contrasting and do not mix pleasantly (e.g. colours of artificial elements)	–	Medium harmony of colours, with one or two dominant tones that do not mix pleasantly (e.g. colours from artificial elements)	–	High colour harmony, presence of subtle colours or balanced colour temperatures
Texture	Grain	Fine grain	–	Medium grain	–	Coarse grain
	Density	Clumped	–	Granular	–	Dotted, Scattered
	Regularity	Random, Patchy	–	Gradational	–	Uniform
Scale	Enclosure	Narrow views 90º −180º	–	Medium views 180º a 270º	–	Wide views >270°
	Extension	Close < 1500 m	–	Mid-range 1500–5000 m	–	Panoramic > 5000 m
Space	Scenic value	Low scenic value. Mundane landscape, absent or not noticeable landscape features.	–	Medium scenic value. Spatial composition includes at least one interesting feature or a focal point.	–	Presence of interesting / rare features or strong focal point; or a canopied scene
	Visual absorption	Landscape with low visual absorption (e.g., the presence of artificial elements dissonant in height in prominent places of the landscape or landscapes with low relief)	–	Landscape with average visual absorption of dissonant elements in the landscape. Some elements or features are not fully framed (e.g., structures built by the water or on plateaus)	–	Landscape with high visual absorption of dissonant elements in the landscape; or without the presence of dissonant elements (e.g., structures are scattered halfway up the slope or against a background that softens their contrast)
	Space harmony	Low harmony/unity between the different elements. No combinations of similar elements, or very few artificial elements adapted to existing natural conditions.	–	Harmony/average unit. Slight combination of similar or related landscape elements; or presence of some artificial elements adapted to the existing natural conditions	–	High harmony/unity between the different elements. A combination of similar or related landscape elements; or most of the artificial elements are perfectly adapted to the existing natural conditions
	Order	No order, random pattern (e.g., typically in more natural landscapes, with low level of human intervention).	–	Medium level of order, some elements are repeated, but they are dispersed in the landscape or not so dominant. Low level of hierarchy at the level of shapes or lines.	–	High spatial order or the presence of elements with repetition, rhythm, direction and gradation (e.g., presence of terraces/terraces with a distinct repetitive pattern)

**Table 3:** Evaluation criteria for the Psychophysical attributes.

Component	Indicator	Evaluation criteria				
		1	2	3	4	5
Complexity	Distribution	Low density or diversity of landscape elements/attributes.	–	Medium density or diversity of landscape elements/attributes.	–	High density or diversity of landscape elements/attributes.
	Spatial Organisation	Low edge density, heterogeneity or aggregation.	–	Medium edge density, heterogeneity or aggregation.	–	High edge density, heterogeneity or aggregation.
	Variation and contrast	Low degree of contrast, low shape or size variation.	–	Medium degree of contrast, medium shape or size variation.	–	High degree of contrast, high shape or size variation.
Historicity	Density of cultural elements	Low density of monuments and historical features (farms, terraces with dry stone walls); or without traditional land uses.	–	Presence of some historical features and traces of the traditional land use pattern, but not dominant in the landscape.	–	High density of monuments, historical features (farms, terraces with dry stone walls).
	Historical continuity	Absent or present, but not noticeable. Recent built structures, last 20 years.	–	Low historical continuity. (e.g. Vineyards PRIDTM 1980)	–	Presence of elements with high historical continuity (e.g. presence of terraces or mortórios, after 1900)
Stewardship	Upkeep / abandonment	Visible or present but not noticeable abandonment of farmland or linear structures.	–	Some agriculture (Vine, olive, almond) with obvious low-maintenance patches (Matos or mortorios); or presence of some ruins or abandoned structures.	–	Good maintenance of linear elements (hedges, walls, paths); or good condition / maintenance of structures, presence of intensive maintenance crops or low abandonment rates.
Imageability	Rarity	Interesting within its setting, but fairly common within the region.	–	Distinctive, though somewhat similar to others within the region.	–	One of a kind; or unusually memorable, or very rare within region.
	Elements	Interesting within its setting, but fairly common within the region.	–	Distinctive, though somewhat similar to others within the region.	–	Consistent chance for exceptional wildlife or wildflower viewing, etc.
Ephemera	Variation	High Seasonal variation. Rich colour combinations, variety or vivid colour; or pleasing contrasts in the soil, rock, vegetation, or water.	–	Medium Seasonal variation. Some intensity or variety in colours and contrast of the soil, rock and vegetation, but not a dominant scenic element.	–	High Seasonal variation. Rich colour combinations, variety or vivid colour; or pleasing contrasts in the soil, rock, vegetation, or water.
	Phenology	Absent or present, but not noticeable.	–	Evidence of some phenology. Observation of few animals and some vegetation in bloom, with seeds or fruits.	–	Spontaneous observation of various animals and vegetation (e.g. bird watching or dominant presence of vines, vegetation in bloom, with seeds, fruits)
Coherence	Spatial arrangement	Low coherence	–	Medium coherence	–	High coherence. Presence of water and correspondence of land form and location of water or vegetation with low fragmentation or repetition of pattern across the landscape
Disturbance	Presence	Several disturbing elements, dominant in the landscape	–	Few or several disturbing elements, but not dominant in the landscape	–	No disturbing elements or not perceptible
	Impact	Large area visually affected by disturbances	–	Some area visually affected by disturbances	–	No area visually affected by disturbances
Naturalness	Vegetation	Low proximity to the natural state	–	Medium proximity to the natural state	–	High proximity to the natural state
	Pattern	Low proximity to the natural state	–	Medium proximity to the natural state	–	High proximity to the natural state
Visual scale	Openness	Low	–	Medium	–	High
	Obstruction	High	–	Medium	–	Low

### Step 2: Evaluation *in situ*

The proposed protocol was tested *in situ*. The major goal of this step is to test its applicability to the study area context and gather evaluation data. Although many studies show no significant differences between field observation and the use of photos, the evaluation *in situ* involves other sensorial dimensions besides vision and allows the discussion of issues of scale and perception before selecting the appropriate photos for respondents to evaluate. Evaluators filled in the protocol by hand about a metre apart, looking at the same landscape from a public access spot with unobstructed views and enabling a good perception of the landscape mosaic. Each indicator was classified from 1 to 5 according to the evaluation criteria, and all the scores were compiled in Excel.

### Step 3: Performance test – standard deviation of scores

In the first phase, we tested the performance of indicators to understand which indicators are more robust. For this, the standard deviation between evaluators' scores was investigated for each indicator. The standard deviation (or σ) is a measure that expresses the degree of dispersion of a data set, showing how dispersed the data is in relation to the mean. Thus, indicators with low standard deviation are desirable since these might point to a tendency to be more straightforward, easier to understand and evaluate. While a higher standard deviation might suggest that an indicator is either harder to measure or the criteria established are inadequate. Hence, these might generate doubts among the respondents; thus, they should be revised or avoided. In this case, indicators in the low performance category were carefully analysed, and the authors considered they should be disregarded. Moderate performance and high performance passed on to the next test – the test of redundancy.

### Step 4: Redundancy test – correlation between indicators

In the next phase, we tested the redundancy of indicators. Since some indicators are somewhat associated with a high degree of correlation, further analysis can help to understand the indicators with lower association (low redundancy) and higher association (high redundancy). If the indicators have a lower association, it generally means that they are highly informative independently and might be interesting to keep. In contrast, indicators with higher association (high redundancy) might be omitted, although their exclusion should be carefully analysed. We may want to keep indicators with high redundancy for normative purposes, for example, in cases where the theoretical basis behind the indicator is proven important for landscape assessment or because the indicators' correlation may change over time; hence it may be kept for monitoring purposes.

**Fig. 2:**
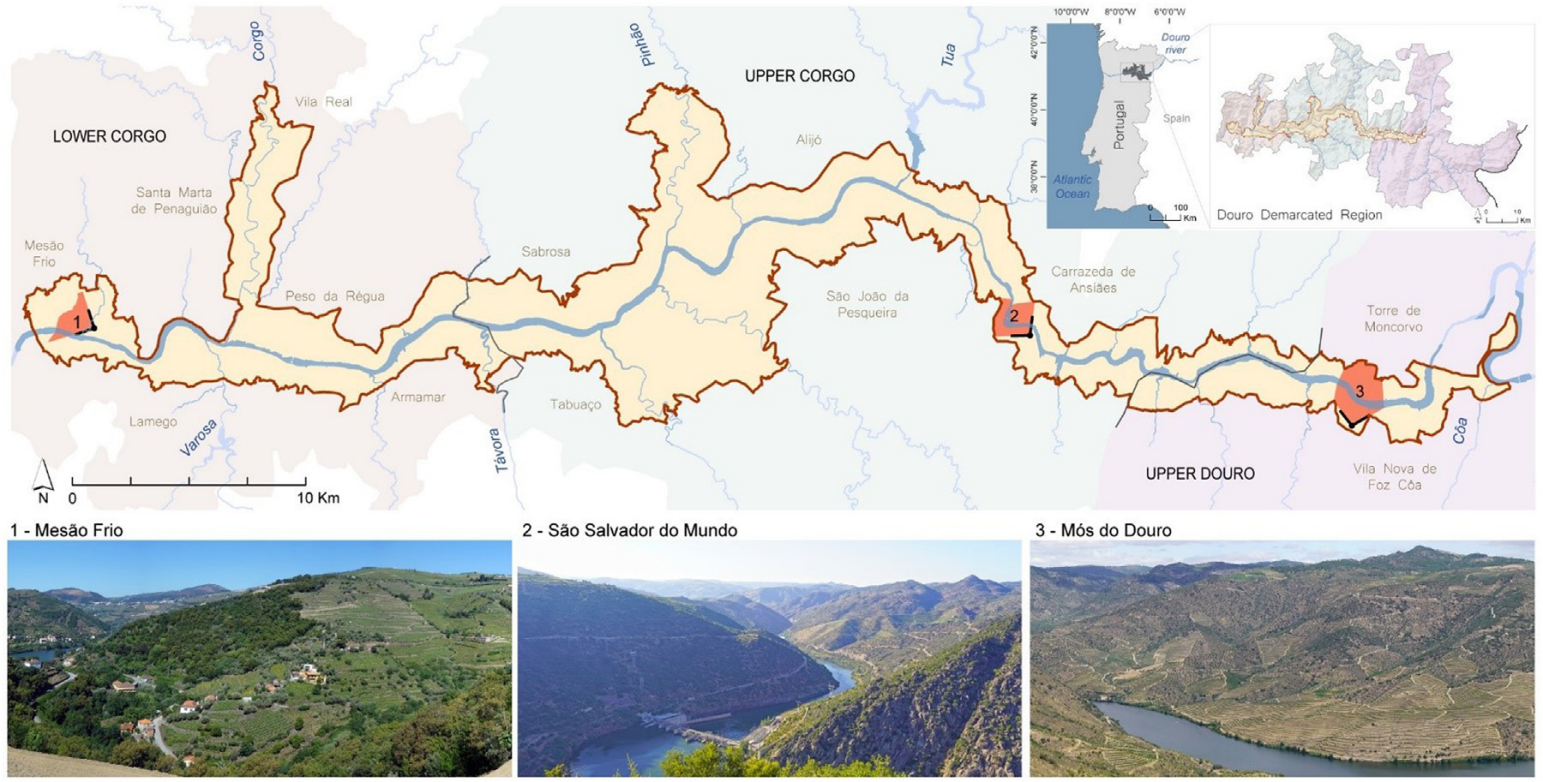
Location of the three visual basins used in this study (1 – Mesão Frio, 2 – São Salvador do Mundo and 3 – Mós do Douro) in the Alto Douro Wine Region (in yellow), its context in the Douro Demarcated Region and mainland Portugal.

**Fig. 3:**
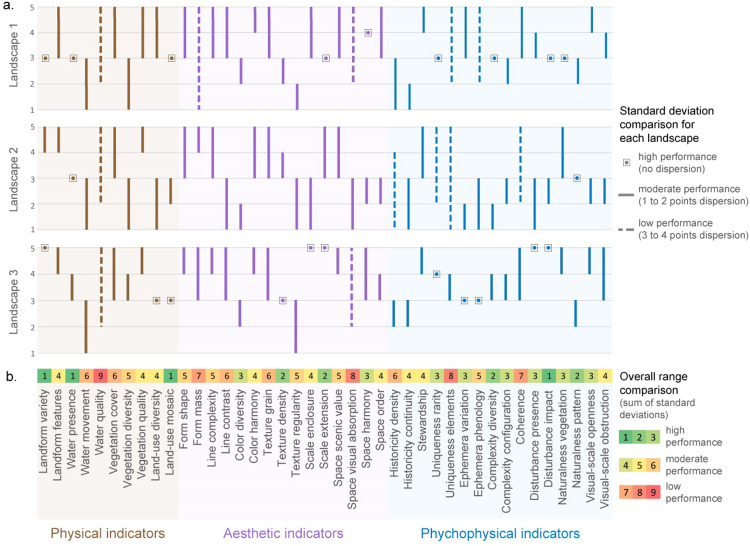
(a) Score range comparison for three landscapes analysed in situ. b) Comparison between the sums of standard deviation for the three landscapes analysed in the field trial. Landscape 1 – Mesão Frio, Landscape 2 – São Salvador do Mundo, Landscape 3 – Mós do Douro.

**Fig. 4:**
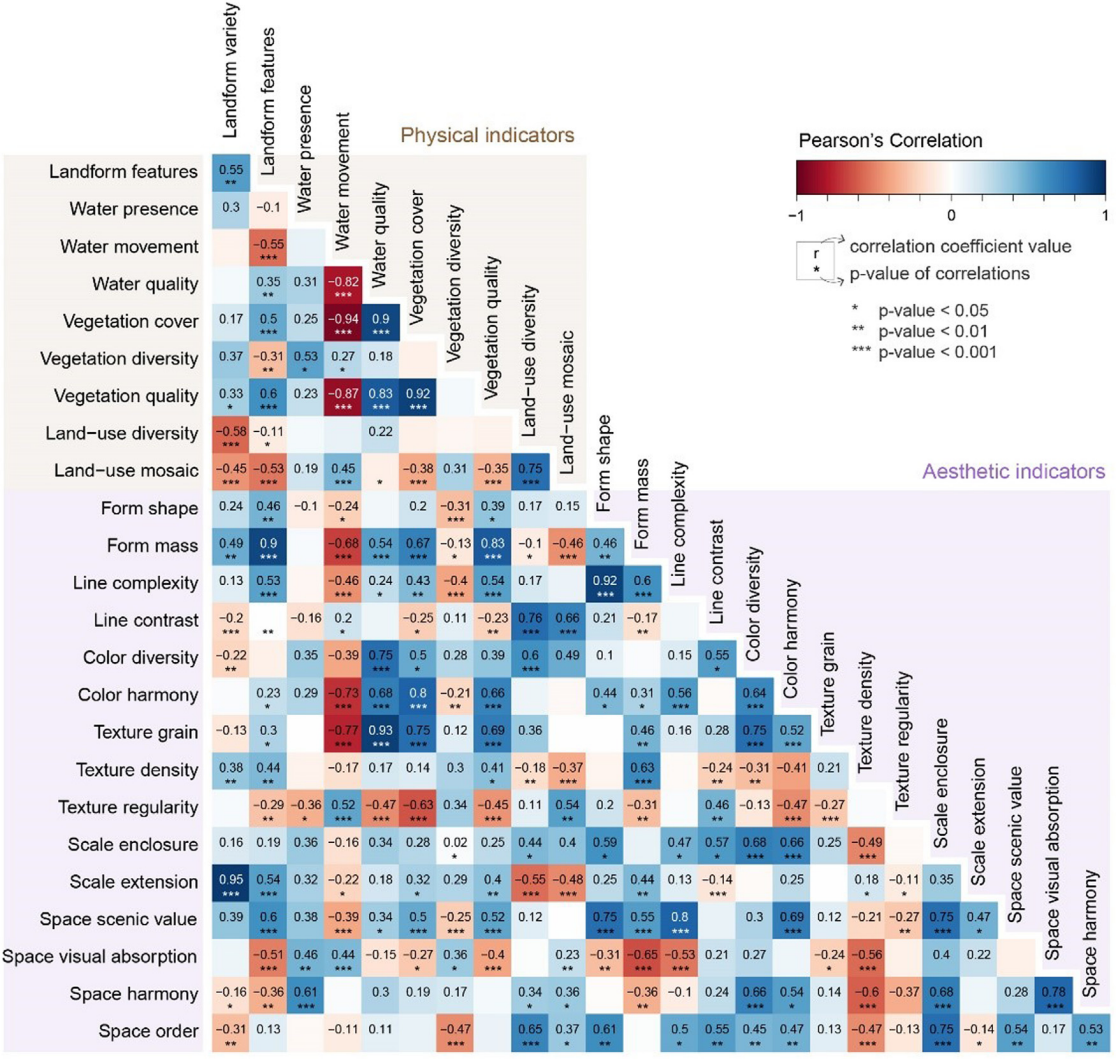
Correlogram between physical and aesthetic indicators.

The correlation was calculated through the pairwise Spearman correlation coefficient, and the analysis was performed in Rstudio with correlograms (packages corrplot and PerformanceAnalytics). The correlation coefficients were analysed through three correlograms between each pair of attributes: physical and aesthetic (Fig. 2), physical and psychophysical (Figure 3), and aesthetic and psychophysical (Fig. 4). The correlation coefficient (or r) was categorised into four classes: i) strong correlation (when *r* ≥ 0.8), ii) moderate correlation (0.5 ≤ *r* < 0.8), iii) weak correlation (0.1 ≤ *r* < 0.5) and iv) not significant (*r* < 0.1, excluded from the correlograms below). The same brackets were considered for negative correlations.

Indicators with higher correlation coefficients (strong and moderate correlations) were carefully analysed and compared so that redundant indicators could be disregarded.

### Step 5: Selection of indicators

Several factors were taken into account in the selection of indicators:

• It was defined that each attribute should have the same number of indicators so attributes all have the same weight in the final score.
• Indicators with low performance for each attribute were disregarded, as they were challenging to rate visually or generated doubts in respondents.
• Pairs of indicators with strong correlations (both positive and negative) were analysed, and only one of these was selected.
• Indicators were reduced to a minimum number, allowing the minimum loss of information while at the same time preserving the representativeness of landscape attributes.
• When in doubt, the indicators' theoretical support and applicability to the study area were carefully considered.

## Method validation

### Context and study area

The proposed protocol was applied at the Alto Douro Wine Region (ADWR) in Portugal, classified by UNESCO in 2001 as a World Heritage with the label of a cultural evolutive living landscape. With over 26 000 ha, it corresponds to a narrow strip along the Douro River showcasing the best-preserved mosaic of the Douro Demarcated Region (RDD). The RDD has three very distinct character zones: Lower Corgo, Upper Corgo and Upper Douro. Their characters differ regarding climatic and topographic conditions, the history of human occupation and landscape mosaic. Hence, three visual basins representative of each character zone were selected (Fig. 2): Landscape 1 - Mesão Frio represents Lower Corgo; Landscape 2 – São Salvador do Mundo represents Upper Corgo; Landscape 3 – Mós do Douro represents Upper Douro.

The assessment of visual quality in ADWR is necessary due to two main factors. The first is related to UNESCO's distinction and Portugal's mission to protect, preserve and monitor the ADWR landscapes. The second is due to the constant change to which these living agricultural landscapes are subject due to various social and economic factors, such as depopulation, economic changes or the design of policies and programs that impact the agricultural sector. One of the main changes in the ADWR landscape has been the expansion of vineyards in areas formerly occupied by forests and Mediterranean scrub. These changes are frequently incompatible with the character of the landscape and the preservation of its natural and cultural values, justifying the need for monitoring.

### Adaptation of the protocol for ADWR

Three evaluators (two landscape architects and one landscape ecologist) applied the proposed protocol *in situ* on separate days in April and July of 2021. The major goals were to test its applicability to the study area context and adapt the proposed protocol to the ADWR landscape, namely the evaluation criteria. For instance, Douro is well known for its hilly landscapes and steep valleys; thus, while evaluating landform, the criteria should be able to reflect this.

Following the LVQPO – PRTest method, the scores obtained and organised in Excel first went through the performance test (Fig. 3). The performance test allowed the discard of five indicators with low performance: water quality, form mass, space visual absorption, uniqueness of elements, and coherence. The indicators with medium performance were also scrutinised taking into account the experience of *in situ* evaluation. Thus, three other indicators were considered potentially tricky to evaluate by future respondents and not robust enough: water movement, ephemera phenology, and space scenic value. Even though these indicators were not at the low performance level, they were found to be very reliant on the expert's knowledge about that particular landscape and not easily translated into the photographic medium.

**Fig. 5:**
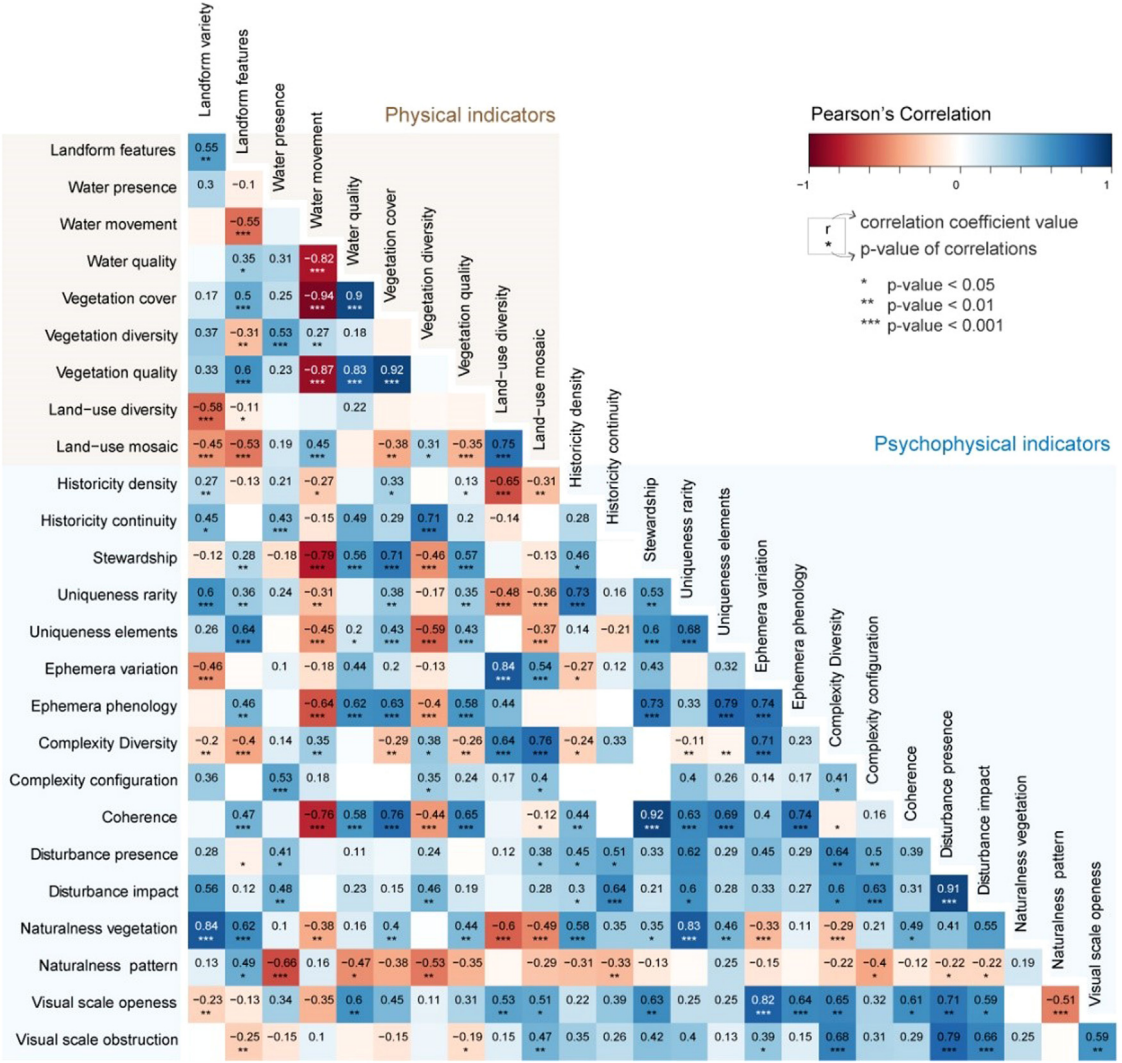
Correlogram between physical and psychophysical indicators.

**Fig. 6:**
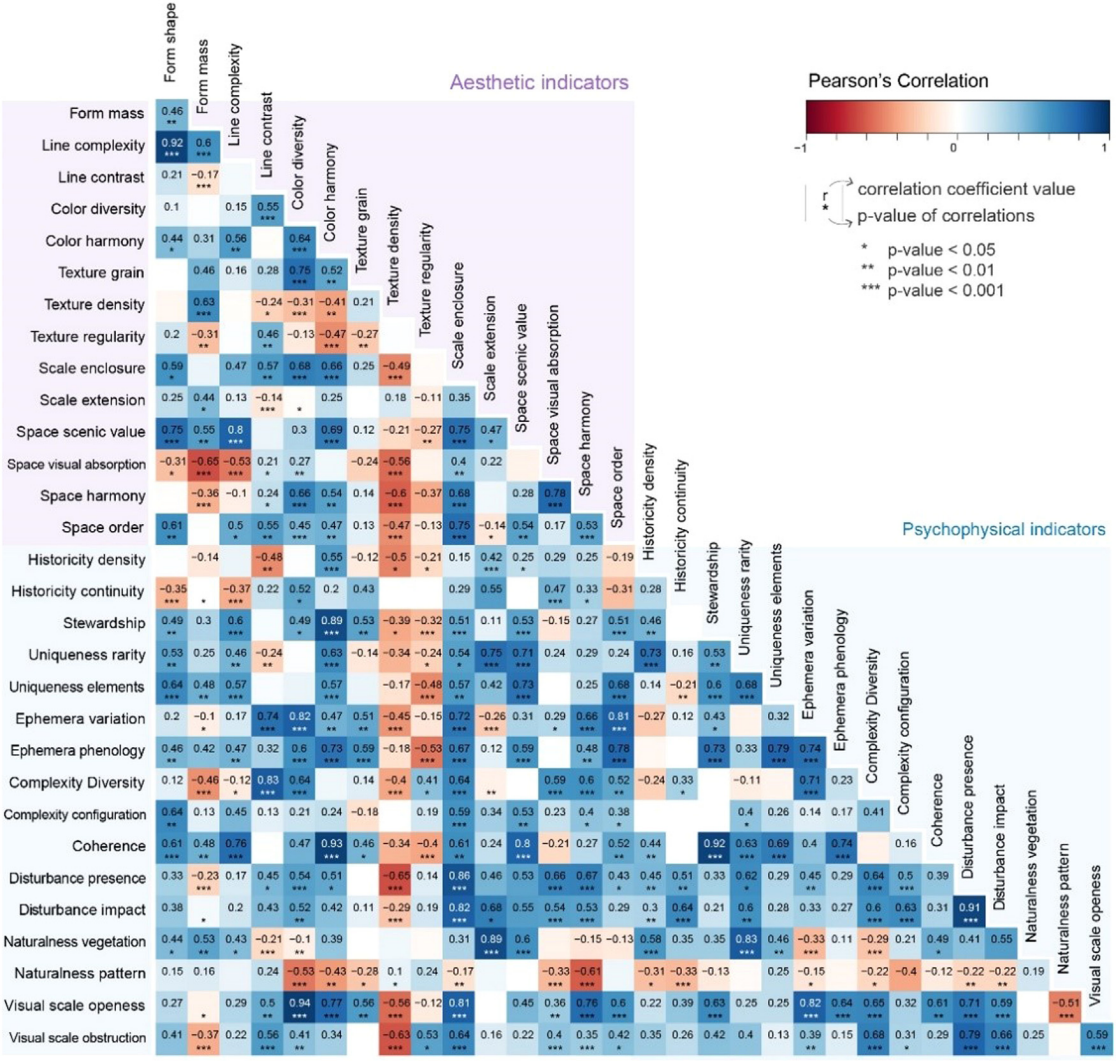
Correlogram between aesthetic and psychophysical indicators.

The next step was the redundancy test, where three correlograms were analysed: a) Physical and Aesthetic (Fig. 4), b) Physical and Psychophysical (Fig. 5) and c) Aesthetic and Psychophysical (Fig. 6). These showcased several high correlations between the indicators proposed. For example, vegetation quality seems to be highly correlated with vegetation cover (*r* = 0.92), but the vegetation cover area is much easier to evaluate than vegetation quality (presence of invasive exotic species). In other cases, the indicators' theoretical support helped to make a decision. For example, the proposed framework had several indicators related to visual scale: scale enclosure, scale extension, visual scale openness and visual scale obstruction. Considering the performance test, scale extension ranked better, but the redundancy test showed a very high correlation of this indicator with landform variety (*r* = 0.95). When the choice was not obvious, the literature on the subject was considered. Since landscape openness is a far more popular indicator and was deemed useful in this study, it was the final choice to represent this landscape component.

**Fig. 7:**
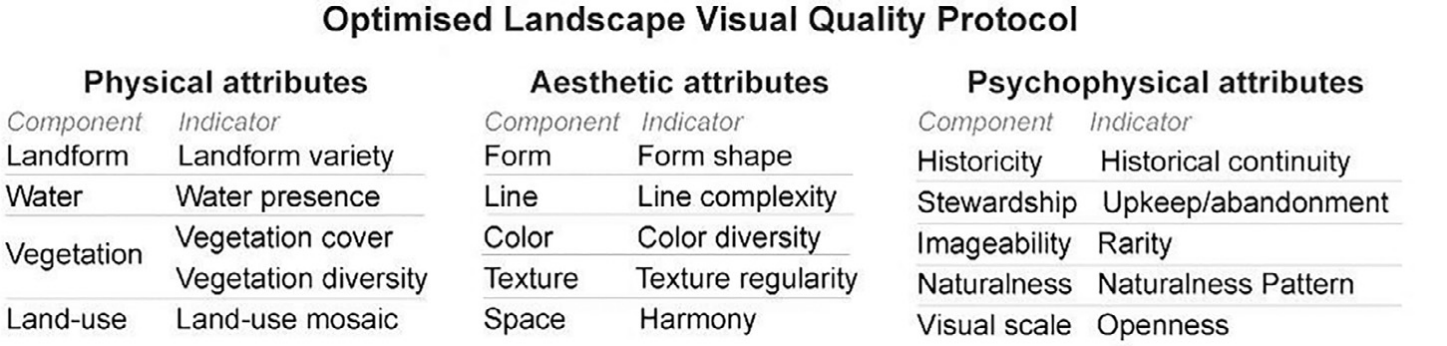
Optimised landscape visual quality protocol.

Ultimately, the LVQPO – PRTest allowed us to reduce the LVQ protocol to 15 robust and pertinent indicators, five for each attribute (Fig. 7). Additionally, evaluation criteria were further adapted to the study area. The resulting optimised protocol was later applied in a photo survey with over 50 respondents, and the indicators and their evaluation criteria were simple and clear to communicate, not generating doubts in terms of comprehension.

There are some limitations to the method that are important to mention. For example, the choice of indicators might not be straightforward after conducting the performance or redundancy tests. In some cases, different indicators might achieve similar results in the tests, not allowing an easy selection between the two. However, the authors see the LVQPO – PRTest as a tool that should always be combined with the judgement about the indicators’ significance in that particular landscape and its importance for the goals of the assessment.

Some shortcomings of the original method (VRM) terminology were also noticed. For example, aesthetic indicators relate only to visual properties and thus could simply be “visual attributes”. Some authors might even argue that aesthetic attributes are psychophysical [8]. In the end, we opted to keep the attributes designations as proposed by the original authors, as these types of discussions do not fit the scope of this paper.

Regarding the method application, the study considers three sites and three evaluators, which is enough for method demonstration purposes, but ideally a larger selection of different sites and more evaluators should be desirable to assure the reliability and transferrability of the study.

Despite the LVQPO – PRTest limitations, the selection of indicators in some studies still seems to rely only on the literature review or practical issues, such as data availability or the goal of assessment. Hence, we believe the methodology presented can be useful for researchers and practitioners. To conclude, the LVQPO – PRTest method does not propose a new method to assess visual quality, but rather an optimisation of the Visual Resource Management Method, including psychophysical attributes proposed by Ode [12] and focussing on the issue of indicator selection. Furthermore, it has the potential to be adaptable to landscape visual studies with different goals and in different landscape contexts.

## CRediT authorship contribution statement

**Ana Medeiros:** Conceptualization, Formal analysis, Writing – original draft. **Cláudia Fernandes:** Conceptualization, Writing – review & editing, Supervision. **João F. Gonçalves:** Conceptualization, Formal analysis, Writing – review & editing. **Paulo Farinha-Marques:** Conceptualization, Writing – review & editing.

## Declaration of Competing Interest

The authors declare that they have no known competing financial interests or personal relationships that could have appeared to influence the work reported in this paper.

## Data Availability

Data will be made available on request.
